# Swarm Intelligence in Animal Groups: When Can a Collective Out-Perform an Expert?

**DOI:** 10.1371/journal.pone.0015505

**Published:** 2010-11-24

**Authors:** Konstantinos V. Katsikopoulos, Andrew J. King

**Affiliations:** Center for Adaptive Behavior and Cognition, Max Planck Institute for Human Development, Berlin, Germany; University of Sheffield, United Kingdom

## Abstract

An important potential advantage of group-living that has been mostly neglected by life scientists is that individuals in animal groups may cope more effectively with unfamiliar situations. Social interaction can provide a solution to a cognitive problem that is not available to single individuals via two potential mechanisms: (i) individuals can aggregate information, thus augmenting their ‘collective cognition’, or (ii) interaction with conspecifics can allow individuals to follow specific ‘leaders’, those experts with information particularly relevant to the decision at hand. However, a-priori, theory-based expectations about which of these decision rules should be preferred are lacking. Using a set of simple models, we present theoretical conditions (involving group size, and diversity of individual information) under which groups should aggregate information, or follow an expert, when faced with a binary choice. We found that, in single-shot decisions, experts are almost always more accurate than the collective across a range of conditions. However, for repeated decisions – where individuals are able to consider the success of previous decision outcomes – the collective's aggregated information is almost always superior. The results improve our understanding of how social animals may process information and make decisions when accuracy is a key component of individual fitness, and provide a solid theoretical framework for future experimental tests where group size, diversity of individual information, and the repeatability of decisions can be measured and manipulated.

## Introduction

The incredible range of sociality that exists in the animal kingdom has intrigued behavioural and evolutionary biologists. There is a rich and varied literature that strives to explain the origins and maintenance of group living [1], and a recent focus has been how social animals choose between alternative actions [2], [3], vital if a group is to remain a cohesive unit and accrue the many advantages of group living [1]. Previous work has considered the time-costs for decisions where groups adopt the action preferred by a single despot, or the action preferred by a majority of group members [4]. However, animals need to also maximise the accuracy of decisions if they are to best exploit potential opportunities and avoid danger. Whilst research has explored how groups choose among a number of actions, e.g., insects [5]; fish [6]; birds [7]; and mammals [8], there has been few *a priori*, *theory-based* expectations about the conditions under which a collective outperforms an expert, or vice versa [9], [10]. With the recent interest in *swarm intelligence* in behavioural and evolutionary ecology, a re-examination of the relationship between the uses of these alternate decision-making rules has been called for [9], and this is our contribution.

We present conditions (involving group size, and diversity of individual information) under which groups would be expected (if they want to maximise accuracy) to adopt one of two decision rules: (i) aggregate information possessed by individuals, thus augmenting their ‘collective cognition’ [9], [11], or (ii) adopting the choice of a single ‘expert’ [12], [13]. In both cases, we assume that information is combined and processed through social interaction, providing a solution to a cognitive problem in a way that cannot be implemented by isolated individuals – a kind of *swarm intelligence* [9], [14]. In both cases the accuracy of decision-making is expected to increase with larger group sizes, as a consequence of pooling information from more individuals, or due to an increased potential for diversity and specialisation of individuals [6], [10], [15]. In all the models we present, although we expect animals to make rational choices between these two possible strategies, we assume that the selection of an aggregated or expert choice rule takes place through an evolutionary process [13], [16]. We choose to model the decisions of groups of 3–15 members, since the improvement in any group-size benefits with respect to aggregated information is expected to diminish with larger group sizes [10]. In addition, within this range of group-sizes, it is plausible that researchers can (a) train individuals (so that the diversity of information within a group can be manipulated) and (b) monitor multiple individuals behaviour simultaneously. It is therefore our hope that our insights can act as a springboard for empirical studies to realistically test our predictions in the laboratory and/or field.

## Results and Discussion

**Figure 1:**
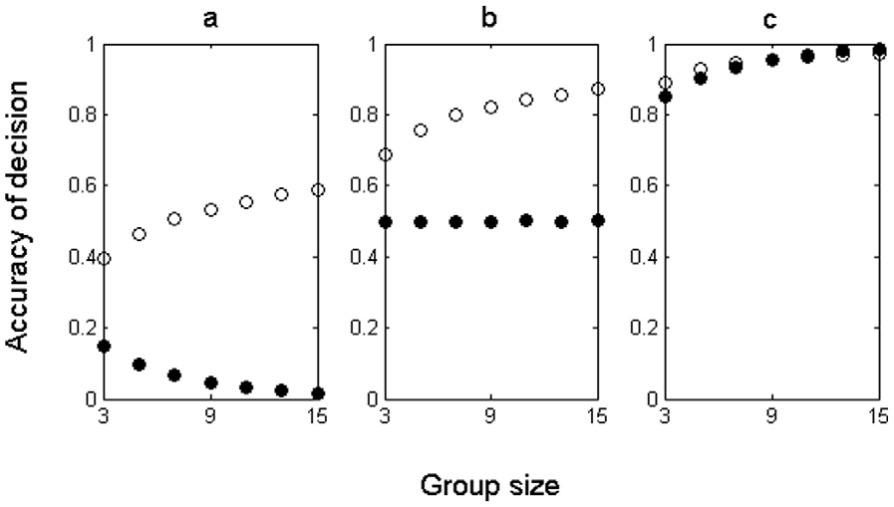
The accuracy of one-shot decisions using an aggregated rule (filled circles) and expert rule (open circles) as a function of group size. **a**. mean individual probability of choosing the correct option, μ = 0.1, with standard deviation, σ = μ/1.96; **b**, μ = 0.5, σ = μ/1.96; **c**, μ = 0.9, σ = μ/1.96. Results presented are the average of 10,000 simulations.

We began by considering a situation in which individuals have to choose between two options, *A* and *B*, for a single decision, where *A* is the correct choice for all individuals (i.e. there is little or no conflict of interests), the level of information (‘accuracy’) individuals possess is variable [17], and sampled from a normal distribution. This is likely to be representative of a variety of binary choices faced by social animals (e.g. the presence or absence of a food resource, or a predator) [10]. Our first model (Model 1: see Methods and Analyses) predicts that when individuals favour the incorrect choice, *B* (are misinformed), or are equally likely to choose *A* or *B* (essentially have very little information), groups should adopt the choice of a single expert, especially in larger groups (Figure 1a; Figure 1b). However, if individuals favour *A* with a high probability (are informed), then our model predicts that the collective is equal in accuracy to the expert (Figure 1c). These basic quantitative predictions about adaptive behaviour in one-shot decisions can now be explored retrospectively with respect to previous findings, and offer a platform for testing our model predictions in future decision-making experiments [18], [19] when used in combination with the model extensions we present below.

In our simple model described above, we assume that there are no differential costs associated with each choice rule [20], [21]. This may be an over-simplification. On the one hand, an expert rule could be quicker than an aggregate choice rule since individuals would need to monitor the choice of a single individual, rather than all group members. Thus, for equal accuracies, an expert rule would be less costly (in terms of time), especially as groups grow larger [22]. On the other hand, if it is difficult to identify an expert, individuals may copy a misinformed individual. Although several animal species have evolved specific signals that advertise the information that they possess [23], and expert leaders can emerge even when individuals do not know how the quality of their information compares with that of others [12], [24], identification of an expert may not always be easy. Where information is correlated with age, size, or dominance [25], [26], identification of an expert may be prone to error, especially where group composition is unstable and opportunity for repeated interaction limited. We therefore re-ran Model 1, with two additional modifications (Model 2: see Methods and Analyses). First, instead of group members being able to always identify the expert, we allowed individuals to copy a pre-specified expert with a given probability. Next, we relaxed this assumption further, assuming in addition to the probability that individuals copied the most informed group member, that there was also a probability that they copied a less informed group member. In both scenarios, we once again found that the expert choice rule outperformed the aggregated rule in terms of accuracy of outcome (Model 2: see Methods and Analyses for full details).

**Figure 2:**
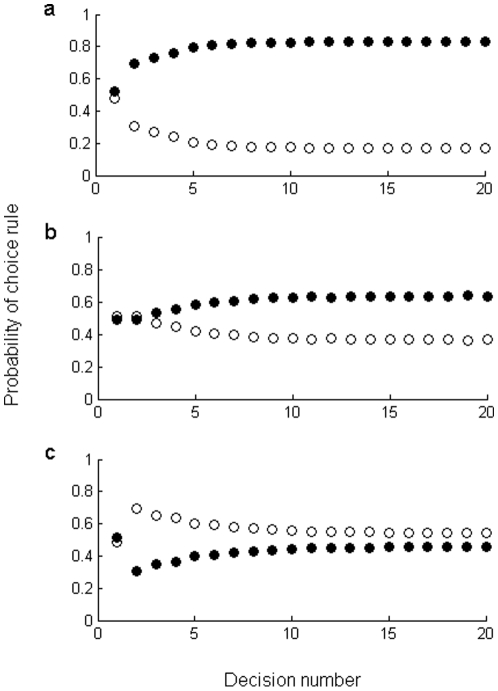
The probablity of usage of the aggregated rule (filled circles) and expert rule (open circles) for repeated decisions, as a function of decision number. **a**, probability that the first decision made is correct = 0.1; **b**, probablity that the first decision made is correct = 0.5; **c**, probablity that the first decision made is correct = 0.9. Results presented are the average of 4,000 simulations (across each group size, *n* = 3, 5, 7, 9, 11, 13, or 15).

Thus far we have reported the results of models that assume single-shot, independent choices. These situations may be representative of ephemeral and/or unstable social groups that are faced with making collective decisions only occasionally (or, more precisely, rarely face repeated collective decisions). In more stable social groups, where individuals encounter repeated collective decisions, individuals may be able to store and recall information [22]. We therefore used a Bayesian model to predict the probability of groups using expert and aggregated rules across time, based on the outcome (accuracy) of past decisions (Model 3: see Methods and Analyses). We assumed that each rule was equally likely to be used to make the first decision, and found that, for all group sizes (*n* = 3 to 15), the probability that groups use each rule-type converges after approximately 20 decisions. The model predicted that the aggregated rule is always favoured, unless the first decision that a group makes is correct with high probability, in which case groups marginally favour the expert rule (Figure 2).

Studies of group decision-making lack testable, well-structured concepts and hypotheses centred on the very thing that is crucial to individual fitness: the accuracy of decisions. Our set of simple models demonstrate that both aggregated and expert rules can enable accurate collective solutions to challenging problems [9], as this has also been found in human group decision-making [27], [28]. Both choice rules we have considered do not necessarily require advanced cognitive abilities, but only that individuals have the potential to acquire information through social interaction, and respond positively to those who possess pertinent information [9], [12] (Models 1 and 2), or update their choice rules based on the success of the previous decisions [29] (Model 3). We explore conditions (heterogeneity and quality of individual information, and group size) under which one would expect ‘follow an expert’, or ‘wisdom of the crowd’ types of choice rules to operate, and have presented explicit, testable predictions. Interestingly, for one-shot decisions, groups should use the information of the most informed individual – the expert – but for repeated decisions it pays to adopt an aggregated rule, as on average, it performs better (see Supplementary Files S1 and S2 for full model outputs). These findings suggest that in ephemeral and unstable social groups, that make collective decisions only occasionally, individuals should follow the most informed individual [12], [30], but in stable social groups that encounter repeated decisions, they would do well to use some aggregate rule [6], [19]. This difference can be attributable to the fact that when using our Bayesian updating process, the group will only continue to adopt an expert choice rule when this is correct with a high probability in the first decision (Model 3, see Methods and Analyses). It also suggests that we might expect selection for appropriate cognitive building blocks [31] in these two types of social systems (ephemeral versus stable groupings). Specifically, whilst each rule requires individuals to strategically respond to others (and thus use social information), in one case this requires identification of informed individuals (expert rule), and in the other, an ability to pool information from multiple individuals (aggregate rule). We now encourage researchers to now test the qualitative and quantitative predictions that we present here, and we believe that an experimental set-up similar to that used by Ward et al. [19] with stickleback fish, *Gasterosteus aculeatusgregarious*, is surely the way to go. Such experimental tests across a variety of taxa will now not only allow us to better understand the evolutionary causes and ecological consequences of social decision-making, but where empirical results fail to match our predictions, this may highlight differences in the costs (information gathering at the expense of basic biological demands), or availability (transmission of inadvertent cues, or intentional signals) of socially acquired information.

## Methods and Analyses

### Model 1. One-shot decisions

We assume a group of *n* = 3, 5, 7, 9, 11, 13, or 15 members (we used odd values of *n* in order to avoid ties), having to choose between two options, *A* and *B*, for a single decision where *A* is the correct choice, and individuals make choices independently of each other (i.e., individual *i*'s choice does not influence individual *j*'s choice) [10], [13]. Each group member has an individual accuracy (personal information, *p_i_*) sampled from a normal distribution with mean μ, and standard deviation σ. Situations where μ = 0.9 represent a scenario where individuals are on the average ‘informed’, μ = 0.5 represents a scenario where individuals have very little information, at ‘chance’ level, and μ = 0.1 is representative of a group of individuals that are on average ‘misinformed’. (We set the deviation of *p_i_*, σ, equal to μ/1.96, so that 95% of samples fell between 0 and 2μ; sampled accuracies that fell outside the (0, 1) interval were not used in the simulation). For μ = 0.5, the distribution of individual accuracies is symmetric, and for μ = 0.1 or 0.9, it is skewed. It has been mathematically proven [32] that aggregated choice rules achieve maximum accuracy if and only if the distribution of individual accuracies is “flat”:
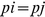
(1)whilst expert choice rules are optimal if and only if the distribution of individual accuracies is “skewed”:
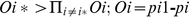
(2)where *i* * is the expert.

### Model 2. One-shot decisions with copying an informed leader

As in Model 1, we assume a group of *n* = 3 to 15 members, which has to choose between a correct and incorrect choice, and one of the group members is an informed leader with accuracy of at least 0.5. The accuracy of the leader was a sample from a normal distribution with mean μ = 0.1, 0.5, or 0.9, and standard deviation σ = μ/1.96 (any sampled leader accuracy outside the (½, 1) interval was not used in the simulation). However, unlike Model 1, where we assume that all individuals can correctly identify and adopt the same choice as the expert on every decision, in this version of the model, we consider that each other group member copies (i.e. makes the same choice with) the leader with a probability *p*, which could equal 0.1, 0.5, or 0.9 (with probability 1−*p*, the group member makes the choice not made by the leader). The copying behaviour of each group member was realised independently of that of the other group members. Leader accuracy was sampled 1,000 times and, for each one of these samples, each group member's copying behaviour was realised 500 times.

We found that the accuracies of both expert (who was the informed leader) and aggregated rules were essentially constant across the mean leader accuracy μ. The accuracies also fluctuated little across group size *n* (the range of accuracy scored predicted was at most 0.1 for the aggregated rule, and at most 0.01 for the expert rule), so we averaged the accuracies across *n*. For *p* = 0.1, the expert rule clearly outperformed the aggregated rule (0.75 vs. 0.27), while the difference decreased for *p* = 0.5 (0.75 vs. 0.58) and there was no difference for *p* = 0.9 (both accuracies equalled 0.75).

In order to further relax the assumption that individuals can always identify the most informed group member, we ran a second simulation of Model 2 with the only difference that there were two potential leaders, (i.e. group members who had accuracy of at least 0.5), and the less accurate leader could also be copied, according to the following scheme: Each other group member copies the most accurate leader with a probability *p* and copies the least accurate leader with a probability *q* (with probability 1−*p*−*q* the group member makes the choice not made by the two leaders if the leaders made the same choice, and makes a random choice if the leaders did not make the same choice). The sum of *p*+*q* could equal 0.1 (for *p* = *q* = 0.05), 0.5 (for *p* = 0.1 and *q* = 0.4, or *p* = 0.4 and *q* = 0.1), or 0.9 (for *p* = 0.1 and *q* = 0.8, *p* = 0.4 and *q* = 0.5, or *p* = 0.7 and *q* = 0.2). We found that the accuracies of both expert (who was the most accurate leader) and aggregated rules were essentially constant across the mean leader accuracy μ. The accuracies also fluctuated little across group size *n* and total probability of imitation *p*+*q* (in both cases, the range of predicted accuracies in decisions were at most 0.15 for the aggregated rule, and at most 0.02 for the expert rule). For all combinations of *n* and *p*+*q*, the expert rule outperformed the aggregated rule, and their average accuracies were 0.83 and 0.75.

### Model 3. Repeated decisions

In order to derive a condition for switching between the expert and aggregated rules for repeated decisions, we first make the following assumption: For each decision, a group uses the expert rule if its optimality condition (equation 2, Model 1) is satisfied; and switch to the aggregated rule if the condition is violated. This assumption is plausible if the aggregated rule is used only when an expert is not optimal, because there may be costs associated with aggregating information from all group members, especially in larger groups [29]. A Bayesian estimate of the accuracy of individual *i*, based on uninformative prior knowledge [32], is:
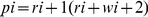
(3)where *r_i_* and *w_i_* are the number of correct and incorrect decisions made by *i*. From (3) and (2), the condition for switching from the expert rule to the aggregated rule turns out to be:
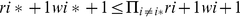
(4)We assumed that it is equally likely that the expert or the aggregated rule is used to make the first decision, and the probability that this decision is correct could equal 0.1, 0.5, or 0.9. Initially, *r_i_ = w_i_* = 0 for all *i*. We simulated the outcome of the first decision, and r_i__*_, w_i__*_, *r_i_* , and *w_i_* were updated (if the expert rule were used, r_i__*_ or w_i__*_ would be set to 1, depending on whether the decision was correct or not; if the aggregated rule were used, *r_i_* or *w_i_* would be set to 1 for all *i*, depending on whether the decision was correct or not). If (4) was satisfied, the aggregated rule would be used in the second decision, and otherwise the expert rule would be used, and so on for the subsequent decisions. For each decision, the accuracy of each individual was its current Bayesian estimate (3).

## Supporting Information

File S1 Output of Model 1. (XLS).

File S2 Output of Model 3. (XLS).

## References

[1] Krause J, Ruxton G (2002).

[2] Conradt L, Roper TJ (2005). Consensus decision making in animals.. Trends in Ecology & Evolution.

[3] King AJ, Cowlishaw G (2009). Leaders, followers, and group decision-making.. Communicative & Integrative Biology.

[4] Conradt L, Roper TJ (2003). Group decision-making in animals.. Nature.

[5] Detrain C, Deneubourg JL (2008). Collective Decision-Making and Foraging Patterns in Ants and Honeybees..

[6] Sumpter DJT, Krause J, James R, Couzin ID, Ward AJW (2008). Consensus Decision Making by Fish.. Current Biology.

[7] Biro D, Sumpter DJT, Meade J, Guilford T (2006). From compromise to leadership in pigeon homing.. Current Biology.

[8] King AJ, Douglas CMS, Huchard E, Isaac NJB, Cowlishaw G (2008). Dominance and Affiliation Mediate Despotism in a Social Primate.. Current Biology.

[9] Krause J, Ruxton GD, Krause S (2010). Swarm intelligence in animals and humans.. Trends in Ecology & Evolution.

[10] King AJ, Cowlishaw G (2007). When to use social information: the advantage of large group size in individual decision making.. Biology Letters.

[11] Couzin I (2007). Collective minds.. Nature.

[12] Couzin ID, Krause J, Franks NR, Levin SA (2005). Effective leadership and decision-making in animal groups on the move.. Nature.

[13] Conradt L, List C (2009). Group decisions in humans and animals: a survey Introduction.. Philosophical Transactions of the Royal Society B-Biological Sciences.

[14] Couzin ID (2009). Collective cognition in animal groups.. Trends in Cognitive Sciences.

[15] Liker A, Bokony V (2009). Larger groups are more successful in innovative problem solving in house sparrows.. Proceedings of the National Academy of Sciences of the United States of America.

[16] Stevens J (2008). The evolutionary biology of decision making..

[17] Rands SA (2010). Self-Improvement for Team-Players: The Effects of Individual Effort on Aggregated Group Information.. PLoS ONE.

[18] Faria JJ, Dyer JRG, Tosh CR, Krause J (2010). Leadership and social information use in human crowds.. Animal Behaviour.

[19] Ward AJW, Sumpter DJT, Couzin LD, Hart PJB, Krause J (2008). Quorum decision-making facilitates information transfer in fish shoals.. Proceedings of the National Academy of Sciences of the United States of America.

[20] Marshall JAR, Dornhaus A, Franks NR, Kovacs T (2006). Noise, cost and speed-accuracy trade-offs: decision making in a decentralized system.. Journal of the Royal Society: Interface.

[21] Trimmer P, Bogacz R, Houston AI, Marshall JAR, McNamara JM (2008). Mammalian choices: combining fast-but-innacurate and slow-but-accurate decision-making systems.. Proceedings of the Royal Society Series B: Biological Sciences.

[22] Dall SRX, Giraldeau LA, Olsson O, McNamara JM, Stephens DW (2005). Information and its use by animals in evolutionary ecology.. Trends in Ecology & Evolution.

[23] Maynard-Smith J, Harper D (2003). Animal Signals.

[24] Dyer JRG, Johansson A, Helbing D, Couzin ID, Krause J (2009). Leadership, consensus decision making and collective behaviour in humans.. Philosophical Transactions of the Royal Society B-Biological Sciences.

[25] King AJ, Johnson DDP, Van Vugt M (2009). The Origins and Evolution of Leadership.. Current Biology.

[26] Van Vugt M, Hogan R, Kaiser RB (2008). Leadership, followership, and evolution - Some lessons from the past.. American Psychologist.

[27] Reimer T, Katsikopoulos KV (2004). The use of recognition in group decision-making.. Cognitive Science.

[28] Hutchinson JMC, Gigerenzer G (2005). Simple heuristics and rules of thumb: Where psychologists and biologists might meet.. Behavioural Processes.

[29] Dall SRX (2005). Defining the concept of public information.. Science.

[30] Reebs SG (2000). Can a minority of informed leaders determine the foraging movements of a fish shoal?. Animal Behaviour.

[31] Stevens JR, King AJ, Hertwig Hoffrage (In Press). The Lives of Others: Social Rationality in Animals.. Simple heuristics in a social world.

[32] Katsikopoulos KV, Martignon L (2006). Naive heuristics for paired comparisons: Some results on their relative accuracy.. Journal of Mathematical Psychology.

